# Sequencing *Medicago truncatula *expressed sequenced tags using 454 Life Sciences technology

**DOI:** 10.1186/1471-2164-7-272

**Published:** 2006-10-24

**Authors:** Foo Cheung, Brian J Haas, Susanne MD Goldberg, Gregory D May, Yongli Xiao, Christopher D Town

**Affiliations:** 1 The Institute for Genomic Research, 9712 Medical Center Drive, Rockville, MD 20850, USA; 2 J. Craig Venter Institute, 9704 Medical Center Drive, Rockville, MD 20850, USA; 3 National Center for Genome Resources, 2935 Rodeo Park Drive East, Santa Fe, NM 87508, USA

## Abstract

**Background:**

In this study, we addressed whether a single 454 Life Science GS20 sequencing run provides new gene discovery from a normalized cDNA library, and whether the short reads produced via this technology are of value in gene structure annotation.

**Results:**

A single 454 GS20 sequencing run on adapter-ligated cDNA, from a normalized cDNA library, generated 292,465 reads that were reduced to 252,384 reads with an average read length of 92 nucleotides after cleaning. After clustering and assembly, a total of 184,599 unique sequences were generated containing over 400 SSRs. The 454 sequences generated hits to more genes than a comparable amount of sequence from MtGI. Although short, the 454 reads are of sufficient length to map to a unique genome location as effectively as longer ESTs produced by conventional sequencing. Functional interpretation of the sequences was carried out by Gene Ontology assignments from matches to *Arabidopsis *and was shown to cover a broad range of GO categories. 53,796 assemblies and singletons (29%) had no match in the existing MtGI. Within the previously unobserved *Medicago *transcripts, thousands had matches in a comprehensive protein database and one or more of the TIGR Plant Gene Indices. Approximately 20% of these novel sequences could be found in the *Medicago *genome sequence. A total of 70,026 reads generated by the 454 technology were mapped to 785 *Medicago *finished BACs using PASA and over 1,000 gene models required modification. In parallel to 454 sequencing, 4,445 5'-prime reads were generated by conventional sequencing using the same library and from the assembled sequences it was shown to contain about 52% full length cDNAs encoding proteins from 50 to over 500 amino acids in length.

**Conclusion:**

Due to the large number of reads afforded by the 454 DNA sequencing technology, it is effective in revealing the expression of transcripts from a broad range of GO categories and contains many rare transcripts in normalized cDNA libraries, although only a limited portion of their sequence is uncovered. As with longer ESTs, 454 reads can be mapped uniquely onto genomic sequence to provide support for, and modifications of, gene predictions.

## Background

In genome projects such as the *Medicago *Sequencing Initiative [1], Expressed Sequenced Tags are of great value for genome annotation because they provide evidence of expression of predicted genes, and by spliced alignment to genomic DNA, they can provide support for gene structures. In instances where genome sequence is not available, EST sequencing provides a first catalog of a species' gene inventory. Combinations of library normalization and deep sequencing are used to maximize gene discovery. Here, we examine whether the deep sequencing made cost-effective by pyrosequencing technology leads to significant new gene discovery in a normalized cDNA library, and whether the short reads produced are of value in gene structure annotation and SSR identification.

454 Life Sciences Corporation, Branford, CT, has developed the first DNA pyrosequencing platform to employ picoliter volumes in a highly multiplexed, flow-through array capable of producing 20–40 million bases per run. Sequencing is performed on randomly fragmented DNA using microbead-based pyrosequencing chemistry. This technology enables sequence data generation for large genome organisms that was previously inaccessible with conventional sequencing platforms because of prohibitive cost and throughput limitations [2]. The usefulness of 454-derived sequences was assessed with the specific goal of identifying new genes and improving gene predictions.

ESTs, are single-pass and partial sequences from cDNA clones that provide a rapid and cost-effective method to analyze transcribed portions of the genome while avoiding the non coding and repetitive DNA that can make up much of the genome of some crop plants. EST sequencing has been shown to accelerate gene discovery including gene family identification [3], large-scale expression analysis [4,5], establishing phylogenetic relationships [6], developing PCR based molecular markers [7], and identifying simple sequence repeats [8] and single nucleotide polymorphisms [9,10]. In both of the finished plant genomes of *Arabidopsis thaliana *and *Oryza sativa*, expressed sequences (ESTs and cDNAs) have been invaluable [11,12] in defining the correct components of gene structure, with spliced alignments of transcript sequences resolving partial or complete exons, splice sites, and in the case of full-length cDNAs, complete gene structures. EST assemblies generated using PASA (Program to Assemble Spliced Alignments) were shown to allow the automated modeling of novel genes and more than 1,000 alternative splicing variations, as well as updates (including UTR annotations) to nearly half of the ~27,000 annotated protein coding genes in the *Arabidopsis *genome [11]. Since experimental biologists are more interested in the reliability of individual predictions than in the average performance of gene predictions, it is important to have an extensive EST collection to guarantee the quality of individual gene models. Although large numbers of ESTs have been generated for many species including barley, rice, maize, sorghum, soybean, *Medicago *and wheat, characterizations of the transcriptomes of these species are likely far from complete.

Here, we examine whether the deep sequencing made cost-effective by the 454 technology leads to significant new gene discovery in a cDNA library by comparisons with The Institute for Genomic Research (TIGR) *Medicago truncatula *Gene Index (MtGI) containing 226,923 high-quality ESTs [13]. We also investigate whether the short reads produced are of value in gene structure annotation by comparisons to high quality automated gene prediction generated by the International *Medicago *Genome Annotation Group (IMGAG) [14].

## Results and discussion

The overall objective of this study was to determine if the 454 technology leads to significant new gene/transcript discovery in a cDNA library, and whether the short reads produced by this technology are of value in gene structure annotation.

Library normalization, 454 derived cDNA sequences, assembly, functional annotation, SSRs and new transcripts/genes discovery will be discussed first, followed by an analysis of genome mapping to determine their ability to validate and update gene structures.

### cDNA library production

An adapter-ligated normalized cDNA population was generated from RNA pooled from four aerial plant tissues (flowers, early seed, late seed, and stems) of *M. truncatula *as described in Materials and Methods. This cDNA (size range 800–2,000 nucleotides) was used to construct both a plasmid library and the 454 library used for 454 sequencing. In order to address the number of full length cDNAs generated from the SMART technology, conventional sequencing was carried out and analyzed. A total of 4,445 5' end reads from conventional sequencing were generated that assembled into 3,619 unique sequences. Of these, 1,916 (~50%) assembled sequences have a protein hit that contains the corresponding start codon and are thus potentially full-length. The top protein matches range between 50 to over 500 amino acids in length and would thus indicate that the predicted open reading frame generated from this library did not have any bias towards long or short protein sequences (Table 1). Approximately 30% of these full length cDNAs do not have a full-length counterpart in the current MtGI.

### 454 sequencing

A single 454 run on this sample generated 292,465 reads with an average length of 99 nucleotides and a total length of 29 Mb. In the previous section, conventional sequencing of the plasmid-based library was discussed with reference to the percentage of full length cDNAs. The remainder of the analysis in this manuscript will focus on 454 sequencing of the normalized (but un-cloned) cDNA population. Conventional sequencing on the plasmid library was performed using a plasmid-located primer close to the 5' end of the cloned cDNA and thus represents the 5' ends of the cDNA population generated by reverse transcription and second strand cDNA synthesis. By contrast, the 454 library preparation involves random shearing of the normalized but un-cloned cDNA population, fragment end polishing, adaptor ligation, library immobilization, fill-in reaction, single stranded DNA library isolation and pyrosequencing [2]. The 454 reads therefore originate from random locations within each cDNA and may have either orientation. However because the original cDNA preparation involved the use of directional adapters that were subsequently used for cloning, we can also recognize the 454 reads that originate from the 5' and 3' ends of the cDNA population by the presence of different adapter sequences and, in the case of the 3' end, also by polyA tracts (both adapter and polyA tracts were removed from the 454 reads before assembly). Searching for the directional adapters resulted in 41,877 reads containing the 5'end adaptor while 50,594 reads either contained the 3'end adaptor or a poly A/T tail. In addition the complete set of reads had matches to over 50% of the *Arabidopsis *proteome. The presence of adapter sequences, poly A tracts and hits to the Arabidopsis proteome indicates that the 454 sequences represents both good coverage both of the ends (presumably UTRs) and within the protein open reading frames.

### 454 cDNA sequence assemblies

Cleaning (removal of adapter, polyA tail, etc) of these sequences resulted in a total of 252,384 high quality reads with an average length of 92 nucleotides totaling 23 Mb. After clustering and assembly using TGICL clustering utilities [15], 101,650 sequence reads were incorporated into 33,865 assemblies leaving 150,734 singletons for a total of 184,599 unique sequences. Most assemblies are short (Table 2) and contain few component reads (Table 3). This is presumably due to both the length of the individual reads and the low coverage of the transcriptome represented in this dataset. Based on the current MtGI, the transcriptome space was estimated as follows: ~40,000 sequences of approximately 2,000 nucleotides equates to 80 Mb of unique *M. truncatula *expressed sequence. The 23 Mb of 454 cDNA sequence analyzed in this study represents only 0.28 times coverage of the transcriptome space, and therefore any attempt at clustering and assembly will not generate high levels of overlapping reads. In order to address the effectiveness of normalization of the library, a comparison was made between the top five most abundant transcripts from the MtGI and the 454 derived sequences. Representation of the most abundant transcripts was 25 to 50 fold reduced in the normalized cDNA population as compared to the collection of ESTs in MtGI, the vast majority of which are derived from non-normalized libraries (Table 4).

### Uniqueness of 454 sequences

Many plant genomes have a high proportion of repetitive sequences, and many multi-gene families. Short reads from recent duplications might not be distinguishable. In order to address this, 454 derived sequences were compared to the MtGI with regard to their ability to map to a single location on the genome. Using a threshold of 95% identity plus 95% coverage, 70% of the 454 unique sequences could be mapped to a unique location. Similarly, 70% of the MtGI sequences could be mapped to a unique location using the same threshold of 95% identity plus 95% coverage. This demonstrates that although short, 454 reads can be mapped to the *Medicago *Genome, with the same confidence as longer ESTs.

### Functional annotation

**Figure 1:**
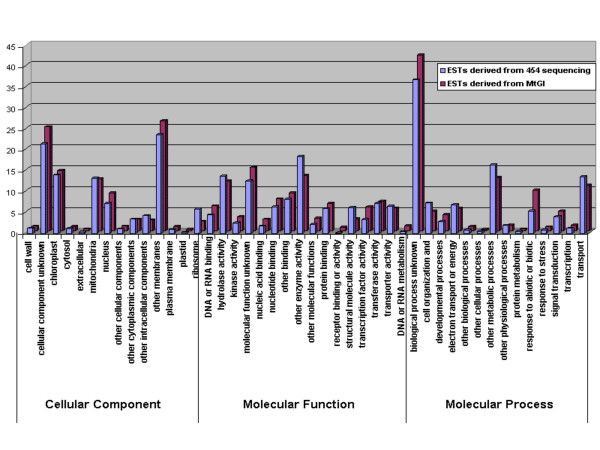
Representation of genome ontology assignments for *M. truncatula *ESTs derived from 454 sequencing and the MtGI.

Gene Ontology (GO) is a controlled vocabulary of functional terms that allows coherent annotation of gene products. In order to assign putative functional roles to the *Medicago *sequences, we used the GO assignments of the *Arabidopsis *proteome [16]. The *Medicago *454 and the MtGI derived unique sequences were compared with the *Arabidopsis *proteome using BlastX (Figure 1). Top protein matches from 12,480 *Arabidopsis *genes was assigned to each of 43,360 *Medicago *454 sequences. Both the MtGI and 454 sequences were plotted side by side and in both cases, the genes cover a broad range of GO categories. Striking differences can be seen within the molecular function of 'response to abiotic or biotic stimulus' and 'biological process unknown as a function'. However this is not surprising since a large percentage of the libraries that were used in the MtGI were generated under abiotic or biotic stress.

### Comparison between 23 Mb of 454 cDNA sequence with 23 Mb of randomly selected EST sequence

In an attempt to compare EST sequence from conventional sequencing and pyrosequencing, we randomly sampled approximately 23 Mb of DNA sequence from the available *Medicago *ESTs in Genbank. Using the same conditions to assemble the 454 cDNA reads and repeating the following analysis three times generates similar results as follows. Cleaning (removal of adapter, polyA tail, etc) of these sequences resulted in a total of 43,985 reads with an average length of 532 nucleotides. After clustering and assembly using TGICL clustering utilities [15], 6,332 assemblies were generated leaving 12,057 singletons for a total of 18,389, unique sequences (Table 5). Not surprisingly, the individual EST reads, and assemblies generated were longer on average and had higher coverage per assembly when comparisons were made with 454 cDNA reads and assemblies (Table 6). Despite the much smaller number of reads, the longer EST length of the individual reads increased the chance of forming contigs and increased the number of reads per assembly. Strictly speaking, an ideal comparison would be to sample EST reads out of the same library from which the 454 cDNA reads were sequenced, however, no similar libraries are publicly available for *M. truncatula *because they were not normalized in the same way.

### Identification of Simple Sequence Repeats (SSRs) Within 454 cDNA sequence assemblies

A total of 401 unique 454 cDNA sequences contained a SSR. Among the SSRs, 143 are trinucleotides, followed by dinucleotides (132), mononucleotides (56) tetranucleotides (47), pentanucleotides (23), and hexanucleotides (8). AG/CT (127) is the most frequent repeat motif, followed by AAG/CTT (86). Among the ESTs without a MtGI hit; 104 sequences contained a SSR. Among these SSRs, 41 are dinucleotides, followed by trinucleotides (30), mononucleotides (10) tetranucleotides (10) and pentanucleotides (10). Randomly selected ESTs from the previous section generated 121 more sequences containing SSRs, three times more sequences with more than one SSR (25) and twice as many SSRs in compound formation. (19) probably due to the longer read and contig lengths.

### Comparison of 454 cDNA aequence assemblies to the TIGR *Medicago *gene index and characterization of novel transcripts

**Figure 2:**
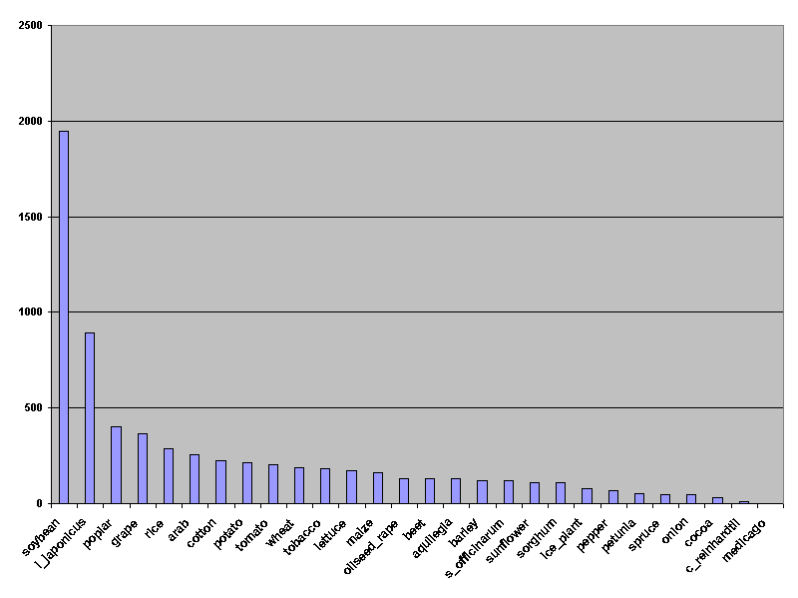
Matches of 53,796 unique sequences without a MtGI hit to other TIGR Plant Gene Indices.

The 454 cDNA assemblies and singletons were searched against the TIGR MtGI, containing 226,923 high-quality ESTs [13]. Approximately 70% of the 454 unique sequences had a blat or a blastn hit to 21,064 MtGI sequences (Expected value cut off 1e-10). From the 53,796 assemblies and singletons (~29%) that had no match in MtGI 3,960 had matches in a comprehensive protein database using blastx (due to the short sequence length, an Expected value cut off 1e-2 was used) and ~2,000 sequences had a blat match to one or more of 27 other TIGR plant gene indices (Figure 2). The ~2,000 reads with matches to TIGR Gene Indices could be condensed into 748 clusters based on their single best match to different parts of the same or orthologous TCs and likely represent this number of previously unobserved *Medicago *transcripts. Similarly, 3,960 sequences that had matches in the comprehensive TIGR protein database could be condensed into 3,144 clusters based on their top protein matches.

**Figure 3:**
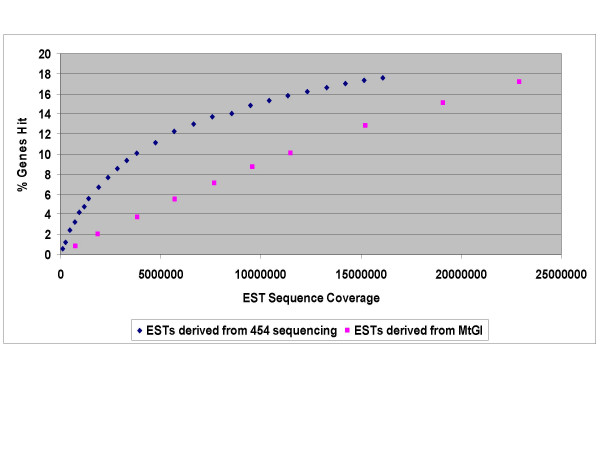
Gene discovery as a function of EST sequencing coverage.

To more clearly demonstrate that the 454 technology could discover new genes by deep sequencing, a comparison was made between the EST sequencing coverage versus the annotated genes of *Medicago *and *Lotus *that are discovered. Figure 3 shows that 454 derived ESTs contain many more gene hits for a given amount of EST sequence coverage when compared to the MtGI. Twice as many gene hits are found up to 750,000 bp of EST sequence coverage and thereafter the rate of gene hits slows but is always found to be substantially higher than the MtGI.

Thus 454 sequencing has revealed many transcripts not previously detected in *Medicago*, some of which have matches in protein or EST databases. This supports the idea that deeper EST sequencing using 454 technology will identify a larger number of expressed sequences than conventional EST sequencing and is effective in revealing the expression of many rare transcripts.

### Mapping novel sequences to *Medicago *genomic DNA

Of the 53,796 sequences not found in MtGI, 13,260 and 9,362 could be mapped using blat with a threshold of 90% identity and 90% coverage and 95% identity and 95% coverage, respectively, to all available *M. truncatula *genome sequence in Genbank (1 April 2006). These transcripts had low levels of repeats sequences [17] and are unlikely to represent sequencing errors, since alignments on the genome using lower thresholds, 60% identity and 60% coverage generated only a 10% increase in matches. At this point, it is estimated that ~50–60% of the *Medicago *euchromatic gene space has been sequenced. Thus the 454 sequences without matches to genomic DNA may be derived from transcripts from the as yet unsequenced euchromatin, from expressed genes residing in the 200+ Mb of heterochromatin or from contaminating nucleic acid e.g. plant bacteria or fungi, although there is no evidence for this last possibility. Thus it is likely that a large fraction of the novel sequences will be useful for gene structure or expression validation in the remainder of the *Medicago *euchromatin or as an indication of the presence of expressed genes in the heterochromatin.

### Use of 454 cDNA reads to support annotation and define exon-intron boundaries

**Figure 4:**
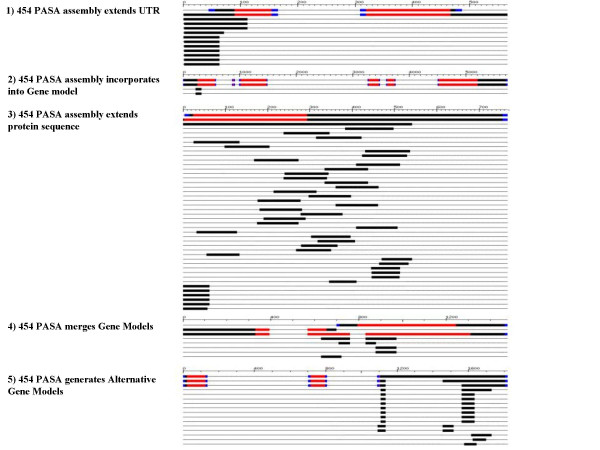
**Examples of annotation updates and alignment assemblies**. The protein coding segments of gene structures (top track) are shown in red and UTRs are shown in black. Alignment of 454 sequences are shown in black below the gene models. Boundaries consistent with the original gene structure annotation are highlighted in blue.

The Program to Assemble Spliced Alignments (PASA) was developed to cluster expressed sequences on genomic DNA using Blat, Sim4 and GeneSeqer and to generate spliced alignments to validate and update gene structures [11]. The gene structure updates provided by alignment assemblies are classified into several distinct categories: UTR extension, gene model incorporation without modification, CDS extension, gene model merging and gene isoform creation (Figure 4).

From the 252,384 sequence reads, a total of 70,026 were mapped to 785 *M. truncatula *GenBank HTGS phase 3 high quality sequence BACs (Table 7), producing 13,933 PASA assemblies. These assemblies were incorporated into 5,124 existing IMGAG gene models. A total of 1,141 assemblies extend 1,061 UTRs, and 278 assemblies alter the protein sequence of 278 genes. Twenty gene models could be merged to produce 10 new genes, and 39 gene model isoforms could be created from PASA assemblies (Table 8). A total of 3,712 PASA assemblies had a [GT, GC]/AG consensus donor/acceptor splice sites at intron boundaries and a near-complete, near-perfect alignment requiring at least 90% of the sequencealigned with at least 95% sequence identity. Introns generated from 454 PASA assemblies of length greater than a generous 2 Kb were excluded, effectively eliminating alignments with false terminal extensionswhich otherwise appear to be high quality alignments.

To determine whether the novel transcripts which lack a MtGI match were useful in *Medicago *gene structure annotation, the 53,796 sequences consisting of assemblies plus singletons that had no match in the existing MtGI were mapped to 785 *M. truncatula *Phase 3 BACs using PASA. From the 10,360 sequences that aligned, 3,221 PASA assemblies were generated and incorporated into 2,186 existing IMGAG gene models. An additional 439 assemblies extend the UTRs of 429 genes and 127 assemblies altered the protein sequence of 127 gene prediction. Five gene models were capable of merging with five other gene models and eight gene model isoforms could be created. A total of 553 of these PASA assemblies conformed to the [GT, GC]/AG consensus donor/acceptor splice sites.

Thus, as with longer ESTs, 454-generated sequences derived from both known and novel transcripts in a cDNA library can be mapped onto genomic sequence and provide valid spliced alignments to provide support for and modifications of gene predictions providing gene structure updates and defining exon-intron boundaries.

## Conclusion

In this study, two major ideas were examined: whether the deep sequencing made cost-effective by the 454 technology leads to significant new gene discovery in a cDNA library, and whether the short reads produced by the 454 technology are of value in gene structure annotation. Approximately 30% of the reads produced by a single 454 run were not found in the *Medicago *Gene Index derived from over 220,000 ESTs from more than 30 libraries, illustrating the power of the deep sequencing facilitated by this technology to generate more gene hits, and reveal rare and novel transcripts, albeit only a small portion of each sequence. Although the read lengths are short, 70 % of the reads were of sufficient length to map to unique locations on the *Medicago *genome as with ESTs from the MtGI via conventional sequencing. Functional annotation shows that the 454 sequences cover a broad range of GO categories. In addition, 454 reads can be mapped onto genomic sequence to provide support for and modifications of gene predictions. We expect that a similar analysis using other plant species would work synergistically with existing EST data and identify new genes/transcripts and/or support a significant number of existing gene models at a very cost effective and efficient manner.

## Methods

### cDNA library construction for conventional sequencing and 454 sequencing

The normalized cDNA population and cDNA plasmid library were constructed employing the Smart cloning methodology [17,18] using the services of Evrogen [19] and Sfi IA/B primers/adapters that permit directional cloning. Reverse transcription was carried out on a pool of RNA from three *Medicago truncatula *tissues (flowers, stems early and late seed) The primer annealing mixture (5 μl) containing 0.3 μg of total RNA; 10 pmol SMART-Sfi IA oligonucleotide (5'-AAGCAGTGGTATCAACGCAGAGTGGCCATTACGGCCrGrGrG-3') and 10 pmol CDS -Sfi IB primer (5'-AAGCAGTGGTATCAACGCAGAGTGGCCGAGGCGGCCd(T)20–3') was heated at 72°C for 2 min and cooled on ice for 2 min. First-strand cDNA synthesis was then initiated by the addition of PowerScript Reverse Transcriptase (BD Biosciences Clontech) in a final volume of 10 μl, containing 1X First-Strand Buffer (50 mM Tris-HCl (pH 8.3); 75 mM KCl; 6 mM MgCl2); 2 mM DTT;1 mM of each dNTP, incubated at 42°C for 1.5 hr and then cooled on ice. The first-strand cDNA was diluted 5 times with TE buffer, heated at 72°C for 7 min and used for amplification by Long-Distance PCR in a 50 μl reaction containing 1 μl diluted first-strand cDNA, 1 × Advantage 2 reaction buffer (BD Biosciences Clontech), 200 μM dNTPs, 0.3 μM SMART PCR primer (5'-AAGCAGTGGTATCAACGCAGAGT-3') and 1 × Advantage 2 Polymerize mix (BD Biosciences Clontech). 18 PCR cycles were performed using the following parameters: 95°C for 7 s; 65°C for 20 s; 72°C for 3 min. Amplified cDNA PCR product was purified using QIAquick PCR Purification Kit (QIAGEN, CA), concentrated by ethanol precipitation and adjusted to a final concentration of 50 ng/μl. For cDNA normalization, 3 μl (about 150 ng) purified ds cDNA plus 1 μl 4× Hybridization Buffer (200 mM HEPES-HCl, pH 8.0; 2 M NaCl) was overlaid with one drop of mineral oil, denatured 95°C for 5 min and then allowed to anneal at 68°C for 4 h. The following preheated reagents were added to the hybridization reaction at 68°C: 3.5 ul milliQ water;1 μl of 5× DNAse buffer (500 mM Tris-HCl, pH 8.0; 50 mM MgCl2, 10 mM DTT);0.5 μl double-strand nuclease (DSN) enzyme. After incubation at 65°C for 30 min., the DSN enzyme was inactivated by heating at 95°C for 7 min. The normalized cDNAs samples were diluted by adding 30 μl milliQ water and used for PCR amplification. The PCR reaction (50 μl) contained 1 μl diluted cDNA; 1 × Advantage 2 reaction buffer (BD Biosciences Clontech); 200 μM dNTPs; 0.3 μM SMART PCR primer; 1 × Advantage 2 Polymerize mix (BD Biosciences Clontech) and was amplified for 18 cycles of 95°C for 7 s; 65°C for 20 s; 72°C for 3 min. One part of the amplified, normalized adapter-ligated cDNA population was digested with SfiI and directionally cloned into Clontech's pDNR vector at the SfiA/B sites.

For 454 sequencing, approximately, 3 μg of the final normalized, adaptor-ligated cDNA population was sheared via nebulization into small fragments a few hundred base pairs in length. The fragment ends were made blunt and short adaptors which provide the priming sequences for both amplification and sequencing of the sample library fragments were ligated onto both ends. These adaptors also provide a sequencing key (a short sequence of four nucleotides) which was used by the system software to recognize legitimate library reads. Next, the library was immobilized onto streptavidin beads, facilitated by a 5' biotin tag on Adaptor B, and any nicks in the double-stranded library are repaired. Finally, the unbound strand of each fragment (with 5'-Adaptor A) was released, and the recovered single-stranded DNA library's quality is assessed. Sequences are available for download [22].

### Sequence analysis

454 cDNA reads were assembled using TIGR Gene Indices clustering tools [14]. Clustering and assemblies from 454 cDNA reads for genome annotation comparisons was carried out using PASA [11].

### Identification and analyses of simple sequence repeats

Perfect dinucleotide to hexanucleotide simple sequence repeats were identified using the MISA [21] Perl scripts, specifying a minimum of six dinucleotide and five tetranucleotide to hexanucleotide repeats and a maximum of 100-nucleotides interruption for compound repeats.

## Authors' contributions

FC conducted the bioinformatics, FC, SMDG, BJH, YX, GDM, CDT contributed to the manuscript writing, SMDG conducted the pyrosequencing, BJH wrote PASA, YX generated the EST data, CDT managed the overall project. All authors read and approved the final manuscript.

## Figures and Tables

**Table 1:** Size distribution of the full length *Medicago *ESTs from convention sequencing using SMART technology

Protein length (amino acids)	0–50	51–100	101–150	151–200	201– 250	251– 300	301–350	351–400	401– 451	400– 501	>501
No of unique sequences	0	45	188	298	320	241	257	194	124	88	161

**Table 2:** Sequence length distribution before and after assembly of sequence reads from a single 454 run of a normalized cDNA library.

**Length Distribution**		
**Nucleotides**	**Reads**	**Post-Assembly**

**< 100**	161,161	113,139
**101–150**	91,203	67,715
**151–200**	19	3,194
**201–250**	1	448
**251–300**		85
**301–400**		16
**401–500**		2
**Total**	252,384	184,599

**Table 3:** Size distribution of 454 assemblies

**Number of reads per assembly**	
**# Reads**	**# Assemblies/Singletons**

**1**	150,734
**2**	20,118
**2–5**	11,209
**6–10**	2,122
**11–20**	369
**21–30**	33
**31–50**	12
**51–100**	2

**Table 4:** Mapping 454 derived ESTs against the top five most abundant TCs in the MtGI

**MtGI id**	**% ests in MtGI**	**% 454 derived ests**	**Annotation**
TC106570	0.53	0.02	RuBisCO small subunit
TC106485	0.47	0.01	Elongation factor-1
TC106598	0.34	0.04	Methionine synthase
TC100296	0.34	0.01	Cytochrome
TC100133	0.33	0.01	Chlorophyll a/b binding protein

**Table 5:** Sequence length distribution before and after assembly of 23 Mb of randomly selected ESTs.

**Length Distribution**		
**Nucleotides**	**Reads**	**Post Assembly**

**100**	12	6
**101–200**	1,717	714
**201–300**	2,475	829
**301–400**	4,802	1,458
**401–500**	8,130	2,373
**501–1000**	26,742	11,518
**1001–2000**	107	1,450
**>2000**		41
**Total**	43,985	18,839

**Table 6:** Size distribution of assemblies produced from 23 Mb of conventional ESTs

**Number of reads per assembly**	
**# Reads**	**# Assemblies**

**1**	12,057
**2**	2,785
**2–5**	2,260
**6–10**	780
**11–20**	321
**21–30**	83
**31–50**	56
**51–100**	38
**>100**	9

**Table 7:** Statistics of PASA alignments of *M. truncatula *454 cDNA reads on finished *Medicago *BACs

Total 454 reads (mapped to genome using blat)	70,026
Valid Blat alignments	40,537
Valid Sim4 alignments	3,643
Total Valid alignments	44,180
Number of assemblies	16,183

**Table 8:** Gene structure updates generated by PASA alignments of 454 cDNA reads on finished *M. truncatula *BACs

**Description**	**# Gene Updates**
EST assembly extends UTRs	1,061
EST assembly alters protein sequence, passes validation	278
EST assembly found capable of merging multiple genes	20
EST assembly stitched into gene model requires alternative splicing isoform	39

## References

[B1] Young ND, Cannon SB, Sato S, Kim D, Cook DR, Town CD, Roe BA, Tabata S (2005). Sequencing the genespaces of *Medicago *truncatula and Lotus japonicus. Plant Physiol.

[B2] Margulies M, Egholm M, Altman WE, Attiya S, Bader JS, Bemben LA, Berka J, Braverman MS, Chen YJ, Chen Z, Dewell SB, Du L, Fierro JM, Gomes XV, Godwin BC, He W, Helgesen S, Ho CH, Irzyk GP, Jando SC, Alenquer ML, Jarvie TP, Jirage KB, Kim JB, Knight JR, Lanza JR, Leamon JH, Lefkowitz SM, Lei M, Li J, Lohman KL, Lu H, Makhijani VB, McDade KE, McKenna MP, Myers EW, Nickerson E, Nobile JR, Plant R, Puc BP, Ronan MT, Roth GT, Sarkis GJ, Simons JF, Simpson JW, Srinivasan M, Tartaro KR, Tomasz A, Vogt KA, Volkmer GA, Wang SH, Wang Y, Weiner MP, Yu P, Begley RF, Rothberg JM (2005). Genome sequencing in microfabricated high-density picolitre reactors. Nature.

[B3] Bourdon V, Naef F, Rao PH, Reuter V, Mok SC, Bosl GJ, Koul S, Murty VV, Kucherlapati RS, Chaganti RS (2002). Genomic and expression analysis of the 12p11–p12 amplicon using EST arrays identifies two novel amplified and overexpressed genes. Cancer Res.

[B4] Ewing RM, Ben Kahla A, Poirot O, Lopez F, Audic S, Claverie JM (1999). Large-scale statistical analyses of rice ESTs reveal correlated patterns of gene expression. Genome Res.

[B5] Ogihara Y, Mochida K, Nemoto Y, Murai K, Yamazaki Y, Shin-I T, Kohara Y (2003). Correlated clustering and virtual display of gene expression patterns in the wheat life cycle by large-scale statistical analyses of expressed sequence tags. Plant J.

[B6] Nishiyama T, Fujita T, Shin-I T, Seki M, Nishide H, Uchiyama I, Kamiya A, Carninci P, Hayashizaki Y, Shinozaki K, Kohara Y, Hasebe M (2003). Comparative genomics of Physcomitrella patens gametophytic transcriptome and Arabidopsis thaliana: implication for land plant evolution. Proc Natl Acad Sci USA.

[B7] Gupta PK, Rustgi S (2004). Molecular markers from the transcribed/expressed region of the genome in higher plants. Funct Integr Genomics.

[B8] Mian MA, Saha MC, Hopkins AA, Wang ZY (2005). Use of tall fescue EST-SSR markers in phylogenetic analysis of cool-season forage grasses. Genome.

[B9] Rafalski A (2002). Applications of single nucleotide polymorphisms in crop genetics. Curr Opin Plant Biol.

[B10] Varshney RK, Thiel T, Stein N, Langridge P, Graner A (2002). In silico analysis on frequency and distribution of microsatellites in ESTs of some cereal species. Cell Mol Biol Lett.

[B11] Haas BJ, Delcher AL, Mount SM, Wortman JR, Smith RK Jr, Hannick LI, Maiti R, Ronning CM, Rusch DB, Town CD, Salzberg SL, White O (2003). Improving the Arabidopsis genome annotation using maximal transcript alignment assemblies. Nucleic Acids Res.

[B12] Yuan Q, Ouyang S, Wang A, Zhu W, Maiti R, Lin H, Hamilton J, Haas B, Sultana R, Cheung F, Wortman J, Buell CR (2005). The Institute for Genomic Research Osa1 rice genome annotation database. Plant Physiol.

[B13] Lee Y, Tsai J, Sunkara S, Karamycheva S, Pertea G, Sultana R, Antonescu V, Chan A, Cheung F, Quackenbush J (2005). The TIGR Gene Indices: clustering and assembling EST and known genes and integration with eukaryotic genomes. Nucleic Acids Res.

[B14] The International *Medicago *Genome Annotation Group. http://www.medicago.org/genome/IMGAG/.

[B15] Pertea G, Huang X, Liang F, Antonescu V, Sultana R, Karamycheva S, Lee Y, White J, Cheung F, Parvizi B, Tsai J, Quackenbush J (2003). TIGR Gene Indices clustering tools (TGICL): a software system for fast clustering of large EST datasets. Bioinformatics.

[B16] The Arabidoposis Information Resource. http://www.arabidopsis.org.

[B17] Ouyang S, Buell CR (2004). The TIGR Plant Repeat Databases: a collective resource for the identification of repetitive sequences in plants. Nucleic Acids Res.

[B18] Zhu YY, Machleder EM, Chenchik A, Li R, Siebert PD (2001). Reverse transcriptase template switching: a SMART approach for full-length cDNA library construction. Biotechniques.

[B19] EVROGEN. http://www.evrogen.com.

[B20] Shagin DA, Rebrikov DV, Kozhemyako VB, Altshuler IM, Shcheglov AS, Zhulidov PA, Bogdanova EA, Staroverov DB, Rasskazov VA, Lukyanov S (2002). A novel method for SNP detection using a new duplex-specific nuclease from crab hepatopancreas. Genome Res.

[B21] Thiel T, Michalek W, Varshney RK, Graner A (2003). Exploiting EST databases for the development and characterization of gene-derived SSR-markers in barley (Hordeum vulgare L.). Theor Appl Genet.

[B22] 454 Sequences. ftp://ftp.tigr.org/pub/data/454.

